# Stabilization and visual analysis of video-recorded sailing sessions

**DOI:** 10.1186/s42492-021-00093-x

**Published:** 2021-10-19

**Authors:** Gijs M. W. Reichert, Marcos Pieras, Ricardo Marroquim, Anna Vilanova

**Affiliations:** 1 Computer Graphics and Visualization, Technical University Delft, Delft, 2628 CD The Netherlands; 2 Visualization Group, Technical University Eindhoven, Eindhoven, AZ 5612 The Netherlands

**Keywords:** Visualization, Sports, Visual-analytics

## Abstract

One common way to aid coaching and seek to improve athletes’ performance is by recording training sessions for posterior analysis. In the case of sailing, coaches record videos from another boat, but usually rely on handheld devices, which may lead to issues with the footage and missing important moments. On the other hand, by autonomously recording the entire session with a fixed camera, the analysis becomes challenging owing to the length of the video and possible stabilization issues. In this work, we aim to facilitate the analysis of such full-session videos by automatically extracting maneuvers and providing a visualization framework to readily locate interesting moments. Moreover, we address issues related to image stability. Finally, an evaluation of the framework points to the benefits of video stabilization in this scenario and an appropriate accuracy of the maneuver detection method.

## Introduction

To assess athletes’ performance during training sessions, in many sports, technology and data analysis are assuming an increasingly larger role. Nevertheless, for sailing, and more specifically, the Olympic dinghy class, such strategies are still not a common practice during training. This is mainly due to the lack of clear strategies for analyzing data obtained from sensors attached to the boats. Therefore, coaches rely mainly on videos to review athletes’ performance. Training sessions usually last 2–3 h, where the coach follows the training boat in a rigid inflatable boat (RIB).

Two maneuvers, known as *tacking* and *jibing*, are the core of the sailors’ strategy to reach the finish line as quickly as possible. The maneuvers allow the boat to advance in a zig-zag manner because it cannot sail straight into the wind. Tacking occurs when a boat turns its bow (front part) against the wind and then keeps turning “through the wind” to catch the wind on the other side of the sail, whereas jibing occurs in the opposite direction. When performing such maneuvers, sailors usually move from one side of the boat to the other (they switch sides).

When and how maneuvers are performed is critical in a race, as every second counts. Hence, they are an important skill to improve and master during training sessions. Coaches therefore record short video clips of the maneuvers, on the order of 1 min, to further discuss them during a debriefing session with the sailors.

However, there is no structured or standard way to register these moments, and most coaches rely on handheld devices for recording, such as their personal smartphones. Because of this, many maneuvers that could be valuable for better analysis of the sailor’s performance are missed.

To avoid the burden of manual capture, coaches have recently switched to cameras that are mounted on their boats and record an entire training session. Despite the clear advantages of relieving the coaches from the recording task and capturing important moments, the task of going through the entire video after a training session (or multiple training sessions) is too costly in terms of time. In addition, with handheld devices, the person registering the footage naturally applies manual stabilization by compensating for the boat’s movement while keeping the subject of interest in focus. On the other hand, this stabilizing effect is lost when the camera is mounted to the trailing boat.

In this work, we address the above-mentioned issues associated with a mounted camera for recording sailing training sessions. Our main contribution is the provision of a pipeline that allows a less time-consuming visual analysis of recorded videos from entire training sessions. It includes stabilization based on the horizon line, extraction of maneuvers from recorded footage, and highlighting potentially interesting segments in time, which can in turn be explored and annotated using a visual interface developed in support of this study. This article is an invited extension of a formerly published conference paper [1] with special focus on various aspects. We have extended the conference paper by adding the stabilization process and its evaluation.

## Related work

Video visualization may assist users in their analysis by removing mechanical tasks, such as viewing the entire footage [2]. Note that the goal is not to provide fully automatic decision-making solutions from video data. Visual tasks related to video visualization are as follows [3]: video annotation, browsing, editing, navigation, recommendation, retrieval, and summarization. In our method, we focus on three of these tasks: annotation, navigation, and summarization. The main goal is to explore interesting events captured on video.

Higuchi et al. [4] proposed a visual analysis tool designed to analyze long sequences with multiple events; however, sailing sessions differ in composition and content from other event-driven activities. We focus on only one complex activity that requires a deeper analysis. A survey by Barris and Button [5] indicated that there is already extensive literature on vision-based analysis related to sports. One common goal is to provide feedback to athletes, as described in ref. [6]. This is also an important aspect with respect to sailing, as videos are used to debrief sailors by reviewing their training session(s) and providing feedback on possible improvements.

Moreover, videos in sports visual analytics may be used passively or actively. In the first case, the video is used to complement other types of data. For instance, Polk et al. [7] used video to reinforce learning outcomes after analyzing tabular data, as one coach noted during their evaluation, “*seeing is believing.*” In the second case, the video is actively used and is considered as the main data input. For example, Legg et al. [8] developed a visual analytics tool for multiple *keyframes* annotation using glyph techniques.

In this study, we examined the importance of video stabilization during the analysis of sailing videos. Video stabilization is a mature area of research, and numerous video stabilization approaches are currently available [9]. Without using other external information, such as inertial sensors, one way to stabilize the video is by using visual cues. In nautical environments, the horizon line is commonly used as a reference for video stabilization [10–12]. These approaches aim to detect the horizon line in the video and transform the video frame to align it horizontally. Other approaches intended to accomplish the same purpose involve separating the image into two regions using machine learning methods [13] or applying pixel-wise segmentation with a fully convolutional network [14]. Another class of methods uses the detection of features around the horizon [15], corner points using an adaptive Harris algorithm [16], or hybrid approaches using feature- and dense- network methods [17]. Our approach focuses on the detection of the horizon line using image-processing techniques.

## Methods

**Fig. 1:**
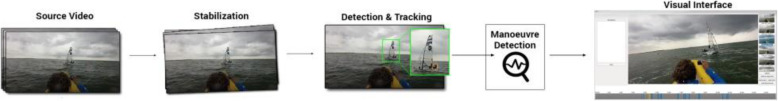
Implemented sailing maneuvers analysis pipeline used to locate maneuvers in a stabilized video and visualize these intervals

To be able to find, extract, and visualize sailing maneuvers, there are a number of necessary steps to follow, hereinafter referred to as the sailing maneuvers analysis pipeline, as illustrated in Fig. 1. These steps are video stabilization, detection and tracking, maneuver detection, and visual analysis.

As mentioned previously, sailors switch sides on the boat during a maneuver. This action can serve as an important visual cue to detect maneuvers from video images. We assume that the video is registered from behind the training boat with the camera facing forward on the chase boat (the RIB). The first step in our pipeline is to stabilize the video in order to compensate for the RIB’s motion on the water. Then, the position of the boat and sailors are tracked, and this information is used to detect maneuvers. Finally, we provide a visualization screen to facilitate the inspection of any detected maneuvers. The goal is to facilitate efficient navigation for the after-training session and allow annotating the most relevant maneuvers.

### Stabilization

For coaching purposes, we consider that it is easier to analyze a stabilized video than its non-stabilized counterpart. In addition, the subsequent stage in the workflow, detecting and tracking the sailors and sailing boat, should benefit from the stabilization as well. Minimizing the motion of a camera capturing the target objects should allow more stable tracking.

Because we only have video data available as input, we must rely on the frames to compute the necessary transformations to compensate for the motion of the camera. The motion of the camera is mainly induced by waves and steering changes. A promising visual cue for deriving induced motion is the horizon line. We assume that the horizon line is always distinguishable in a maritime environment. In addition, we suppose that the horizon line is a long straight line that is not affected by the camera’s lens deformation. After locating the horizon line and using the angle formed by that line with a straight horizontal line, the frame is transformed and therefore stabilized.

**Fig. 2:**
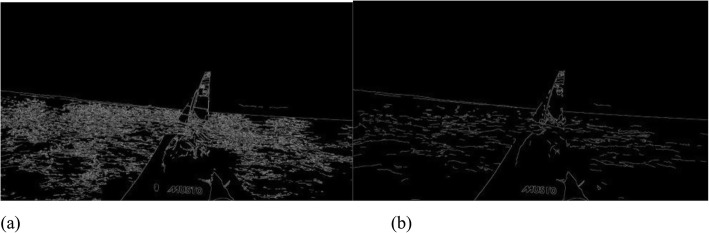
Denoising a video frame using median blur. **a**: Result of Canny edge detection without denoising the frame; **b**: Result after denoising followed by the edge detection algorithm

The first steps of the stabilization process consist of denoising the frames using a median blur filter, followed by edge detection using the Canny edge detector method [18]. The main purpose of denoising is to eliminate spurious edges caused by waves and, consequently, facilitate horizon detection. The differences between not applying and applying denoising are depicted in Fig. 2.

Detecting the horizon line from the edge image is performed in three steps: dilation, detection, and selection. The detected edges are dilated to increase the probability of detecting the horizon. We empirically found that using a 7 × 7 kernel resulted in stable detection. Dilation helps to generate more prominent continuous lines that are easier to select by the voting system used in the horizon-line detection algorithm.

**Fig. 3:**
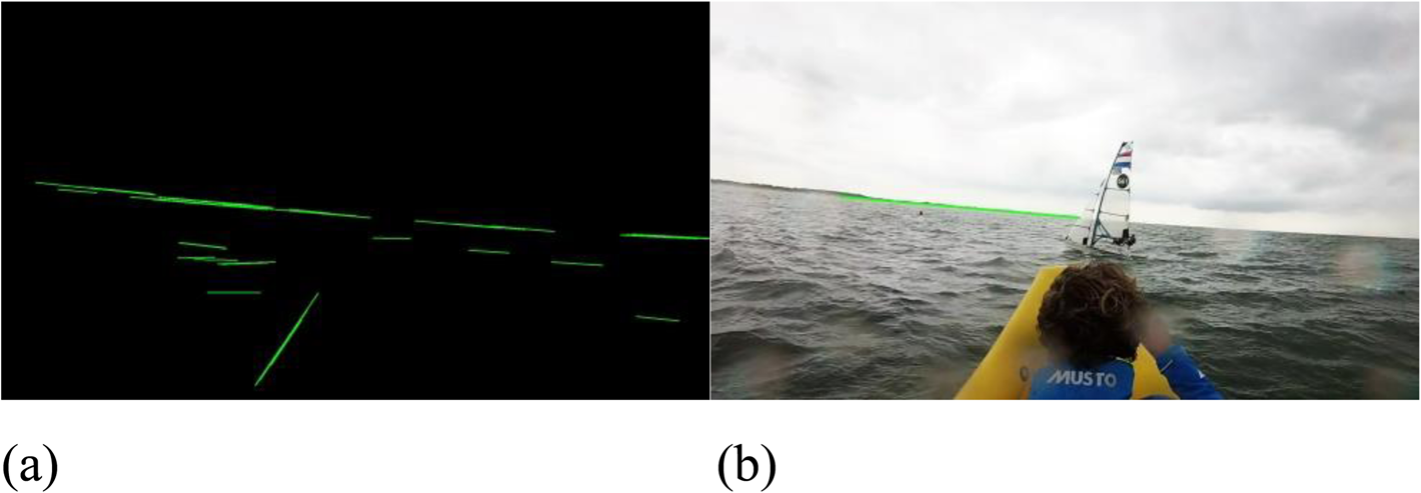
Output of the PPHT and the line used as the horizon. **a**: Lines detected after applying the line detection algorithm; **b**: Example of horizon line obtained from (**a**)

After applying this transformation, the next step consists of detecting and extracting candidate line segments in the dilated image using the Hough transform algorithm. To avoid the high computational cost of the original Hough transform, we use the progressive probabilistic Hough transform (PPHT) algorithm [19], which is a computationally less expensive variant (Fig. 3). The settings used for this algorithm were those from the original paper: *θ* = 0*.*01 and *ρ* = 1. The minimum line segment length limit was 100 pixels, and a voting limit of 150 votes was used during the experiments to avoid selecting small lines caused by water reflections. This parameter is resolution-dependent, and changes linearly with the resolution.

**Fig. 4:**
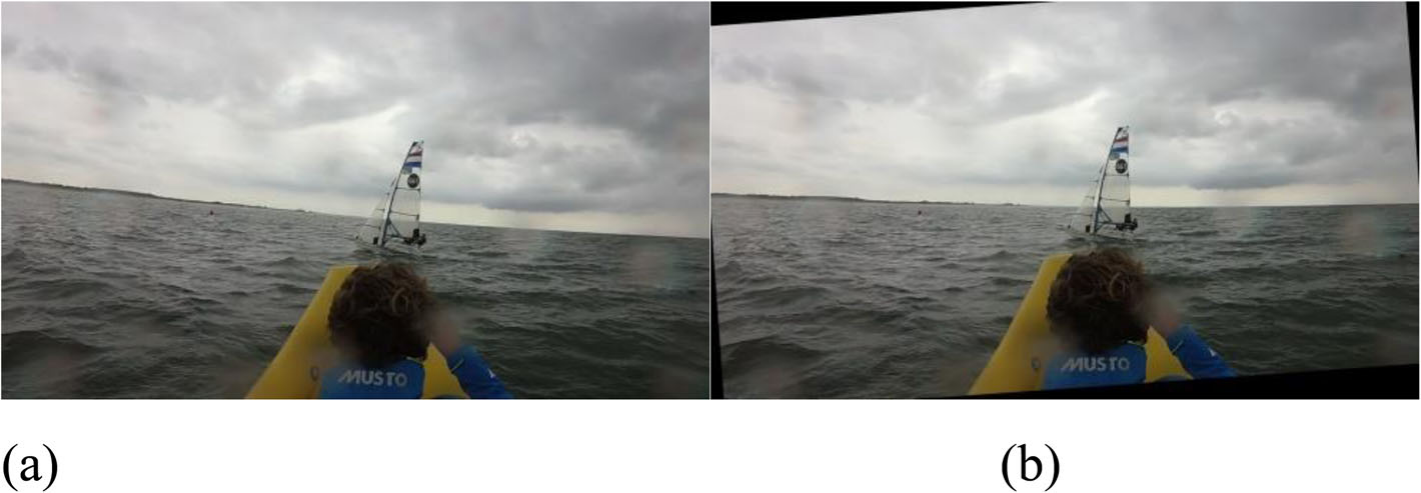
Example transformation of a video frame. **a**: Raw frame extracted from the video; **b**: Result after applying stabilization

The line detector algorithm detects a set of line candidates in a frame. The longest horizontal line segment detected is considered to be the visible horizon. That is, the candidate line segment with the largest absolute horizontal difference (in pixels) between endpoints is selected as the horizon line. The transformations for every frame are then calculated using a previously detected horizon line. The translations and rotations that transform the selected segment to the target destination — the horizon as a horizontal straight line — are computed and stacked in a data structure. This data structure is then filtered using a moving average filter to reduce jitter caused by small differences in rotation and translation per frame, and the transformations are then applied to the corresponding original frames. An example of a source and a transformed video frame using the presented stabilization method is shown in Fig. 4.

### Detecting and tracking the boat and sailors

A maneuver is detected by analyzing the location of the sailor(s) with respect to the boat. To detect boats and sailors, we use a pre-trained neural network for object detection. More specifically, MobileNet [20] was selected, given its low computational cost. The pre-training was executed on the Microsoft Common Objects in Context dataset [21], which already contains images for the classes *Person* and *Boat*.

For each video frame, we expect the network to provide bounding boxes for the located boat and sailors. Notwithstanding, we noted that it fails to detect them in many frames, especially the sailors. We therefore combined the detection network with a tracking method. We used the discriminative correlation filter with channel and spatial reliability as the tracking method [22]. The tracking algorithm is initialized with a bounding box found in one of the frames by the detection network. However, the tracking algorithm is also not perfect and tends to result in drift over time due to accumulated errors. To overcome this issue, the bounding boxes between both methods are compared, and when the difference is above a given threshold, the tracker is re-initialized. In our experiments, we used a threshold of 25 pixels for 1280 × 720 footage images.

If the network fails to detect the boat or sailor, we must rely on the tracker estimation for these frames. Finally, if both methods fail, we assume that any objects are either too far away or are outside the frame. During the experiments, we disabled the tracker whenever the network did not detect a boat or person for more than 60 sequential frames, which empirically avoided most cases of unreliable location data. The combined network and tracking method increased detection accuracy in stabilized videos by 10%–15%.

### Maneuver detection

As mentioned above, we use the action of sailors switching sides to detect maneuvers. We rely on the bounding boxes of the boat and sailors to identify this action. Hence, a signed distance *d* between the center of the boat’s and the sailors’ bounding boxes is computed. Note that only the horizontal difference is considered because we assume that the video is registered from behind the boat. Even if the boat is tilted to one side, the sailors should still be far enough away from the middle of the boat in the video. For dinghy class, this is especially true because sailors lean out over the side of the boat (hike) to keep the boat as vertical as possible.

**Fig. 5:**
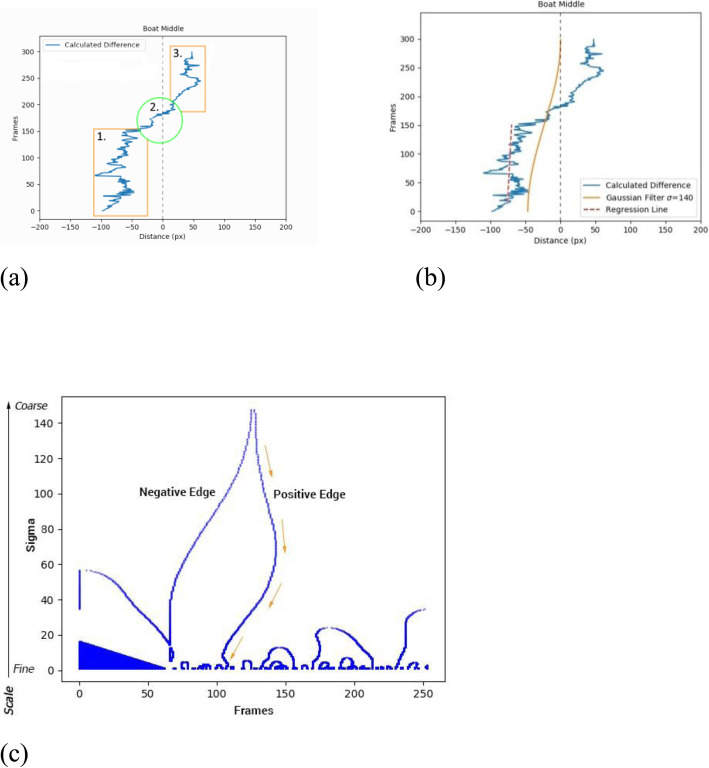
Tracking plots involved in maneuver detection. **a**: Segments of a sailing maneuver. (1) Sailors stable on left side, (2) crossing the middle of the boat, and (3) stable on the right side; **b**: Regression line before zero crossing. Orange line is the output of the (too) coarsely filtered calculated difference; **c**: Adaptation to frames using edge focusing signature graph at different values of *σ*, allowing tracking from coarse to fine

Even though the signed distance *d* contains noise present in the frames, the crossing moment is usually clear under visual inspection, as illustrated in Fig. 5(a). Because we assume that the video is registered from behind the boat, and the boat’s bounding box encloses the whole boat instance, the middle of the bounding box represents the middle of the boat. We define the middle of the boat as a vertical line in the middle of the bounding box. A maneuver is detected whenever the sailor crosses this line and remains on the other side. There are three important moments, as shown in Fig. 5(a). First, we look for the moment when the sailors start to move to the other side. This is followed by the actual crossing. Finally, the maneuver is concluded when the sailors remain on the other side for a sufficient amount of time.

In some cases, noise can nevertheless prevent the stable detection of these moments. A possible approach to remove noise is the use of a sliding smoothing window. In this case, the window size and smoothing parameter *σ* must be defined. Figure 5(b) illustrates this method when *σ* = 140. Even though the noise is removed, the actual crossing moment is offset by more than 50 frames from the real crossing moment. Moreover, to avoid manually defining *σ*, we resort to an adaptation of a scale space method called edge focusing [23]. This allows for more robust location of the crossing moment, as shown in Fig. 5(c).

In our adaptation, we first compute a Laplacian of Gaussian (LoG) filter with *σ* in the range [e^*a*^*,* e^*b*^]*, a* = 5*, b* = 0, using a step size of 0*.*005, as suggested by Haar Romeny [24]. Using the LoG filter for all *σ* values in this range, the zero-crossing frames are stored as the ‘signatures,’ as depicted in Fig. 5(c). The small step size guarantees that negative and positive edges do not last longer than one frame. The positive edge can then be tracked in a coarse-to-fine manner to determine the precise crossing moment, as indicated by the orange arrows in Fig. 5(c).

Finally, we need to determine if the sailors remain long enough on the other side to consider it as an actual maneuver. For this purpose, we compute a regression line [25] on the location data to remove noise from the detection. More specifically, we fit the line to a window that represents the average time to perform half a maneuver. For 30 Hz videos, this implies approximately 90 sequential frames. When the slope of the regressed line is below 0*.*15 rad, we judge the location of the sailors to be stable. Likewise, when the slope of the line increases, we assume that the sailors are starting a maneuver. The slope of the line gradually decreases once they have crossed to the other side, marking the end of the maneuver. We consider the last stable frame before and the first stable frame after the crossing as the maneuver interval.

### Visual analysis interface

**Fig. 6:**
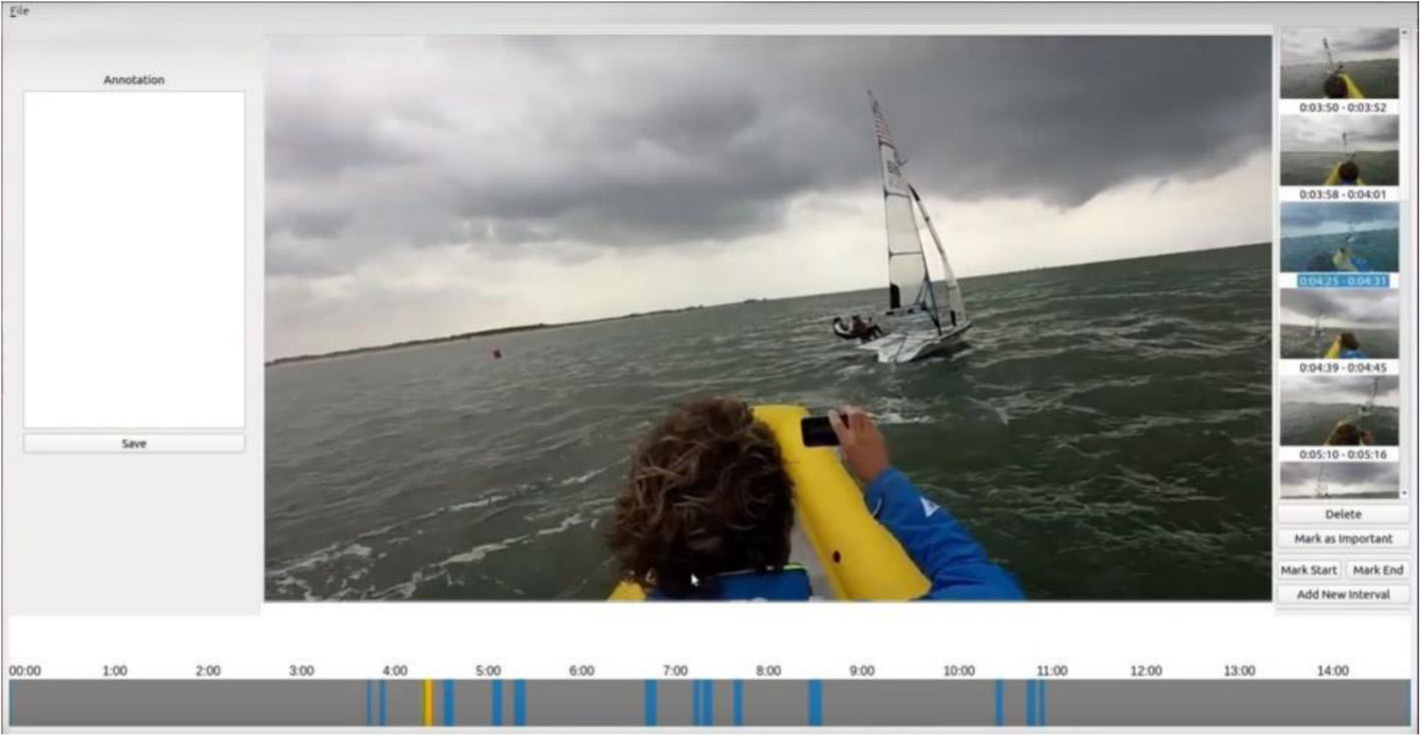
The implemented sailing maneuver analysis pipeline to locate maneuvers in a video and visualize these intervals. The timeline (bottom part) shows in blue the detected maneuvers and in orange the current selected instant in the main window

The last stage of our pipeline is a visual analysis interface that enables exploration of a registered session containing detected maneuvers, as illustrated in Fig. 6. The user has the option to watch the original or stabilized video, depending on his/her preference. The timeline at the bottom highlights the detected maneuvers time intervals in blue and the currently selected maneuver in orange. Alternatively, it is also possible to browse maneuvers using the thumbnails on the right side, where each one is labeled with timestamps marking the beginning and end of the interval. Both navigation options are linked; hence, selecting the interval in the timeline highlights the thumbnail and vice-versa.

We also provide a few tools that may aid a debriefing session. Users can include and remove intervals, mark intervals as important, and annotate selected intervals using the text-box on the left-hand side of the screen. While the timeline with contrasting colors gives a good overview of the potentially interesting events during the session, the thumbnails give an indication of what is occurring during a particular time interval. This facilitates the selection of interesting intervals and the removal of false positives generated by the automatic detection method.

## Results and discussion

In this section, we explain the evaluation process and its outcomes. To evaluate the developed framework, a user study was conducted with seven Olympic-level sailing coaches. We divided the evaluation into several modules: video stabilization, maneuver detection, and general application.

### Stabilization evaluation

To evaluate stabilization, we used both qualitative and quantitative measures based on defined metrics. To objectively quantify the difference between the original video and the result of our stabilization method, we used the peak signal-to-noise ratio (PSNR). The assumption behind this metric is that if the transitions between consecutive frames are smooth, the similarity between consecutive frames is higher. This measurement is defined in decibels between consecutive frames as

1 $$ PSNR=10\;{\mathit{\log}}_{10}\;\left(\frac{{{\mathit{\operatorname{MAX}}}_I}^2}{MSE}\right) $$

where MAX_*I*_ is the maximum intensity that a pixel can have in image *I*. The mean-squared error (*MSE*) for consecutive frames with dimensions *N* × *M* is defined as

2 $$ MSE\;(n)=\frac{1}{MN}\;\sum \limits_{j=0}^M\sum \limits_{i=0}^N{\left[{I}_n\;\left(i,j\right)-{I}_{n+1}\;\left(i,j\right)\right]}^2 $$

**Fig. 7:**
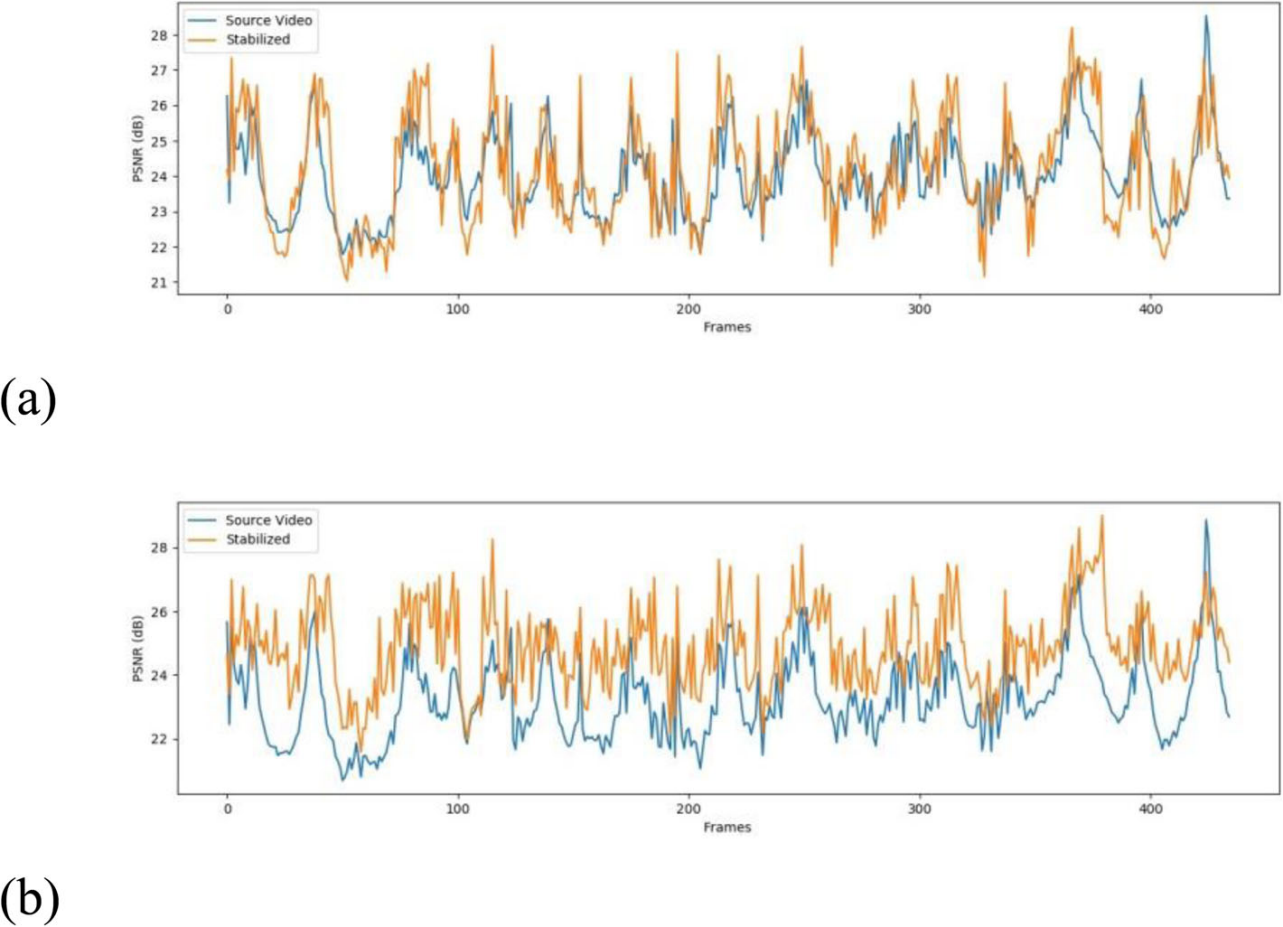
Graphs of non-cropped and cropped PSNR values from a test video. **a**: PSNR graph for the source and stabilized video; **b**: PSNR graph for source and stabilized video with cropped frames

Figure 7(a) indicates that there is little difference between the source and its stabilized counterpart. However, this is mostly caused by the black patches that are used to fill the frame after applying the transformations to a frame in the stabilization method. If the same video is cropped to remove the black patches, as shown in Fig. 7(b), it is clearly apparent that the PSNR of the stabilized video is higher than that of its non-stabilized counterpart. These PSNR graphs can be summarized into a single value by averaging the PSNR for consecutive frames; the result is called the interframe transformation fidelity (ITF). Some experimental results using ITF on four videos taken under different conditions are shown in Table 1.

**Table 1:** Values of ITF for videos under different conditions

Settings	ITF source (dB)	ITF stabilized (dB)
Sunny & Calm	26.58	28.00
Large Waves &Rain	30.05	30.62
Medium Waves &Cloudy	23.23	24.88
Large Waves &Cloudy	30.78	30.98

The results from the quality measures suggest that the stabilized videos are slightly better, and we assume that these should be easier to analyze by a coach. To evaluate this assumption, seven coaches were shown three pairs of videos, each pair consisting of the original video and the stabilized version. Each example was recorded under different weather conditions: cloudy and medium waves, overcast conditions and large waves, and sunny weather under relatively calm seas. Later, for each pair of videos, we asked the following question, where video A was the original video and video B was the stabilized video:

“*Which video (A or B) is easier to analyze?*

1. *Strong preference for video A;*
2. *Maybe video A;*
3. *Both videos are equally easy/difficult;*
4. *Maybe video B;*
5. *Strong preference for video B*

**Fig. 8:**
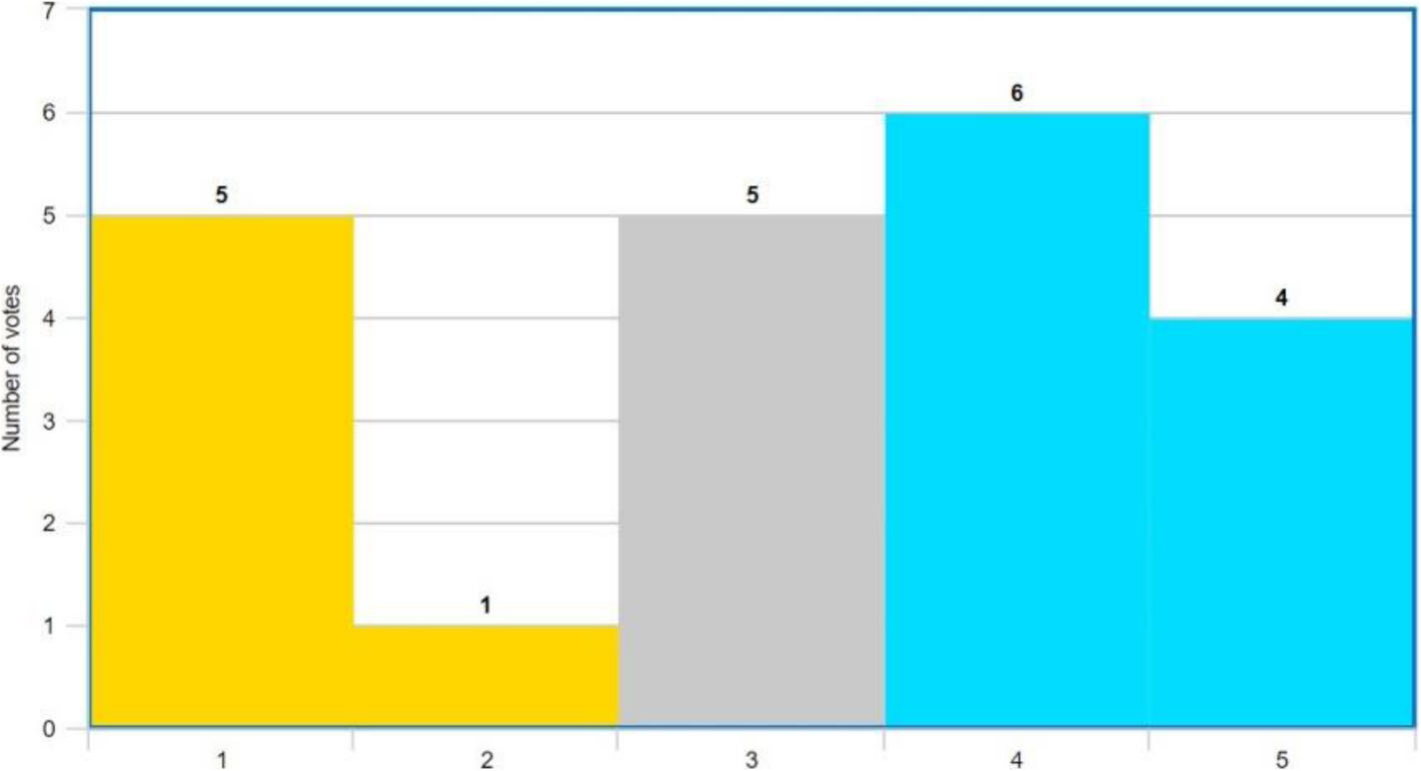
Outcome of the survey question. The results show the 5-point Likert scale results (blue: favored the stabilized version; yellow: favored the original version; gray: neutral opinion)

Each coach was then asked to vote on the three pairs of videos using a 5-point Likert scale. The results are shown in Fig. 8. Based on these results, we can conclude that the stabilized version was preferred, as 10 out of 21 votes (blue background) were in favor of the stabilized videos and the non-stabilized version was preferred only six times (yellow background). Motivations given for these choices in favor of the stabilized versions are that it makes looking at the details easier and that “the movements of the video are caused by the RIB, which are totally irrelevant.” The coaches who were strongly against the stabilized videos stated that the moving edges were too distracting. This problem could be addressed by cropping the video so that no black patches can be seen, or by using in-painting methods to fill in the missing regions.

### Maneuver detection evaluation

For the evaluation, we selected three videos where our assumptions were mostly satisfied (the sailing boat was followed from behind and was in full view of the camera). We removed situations in which these assumptions did not hold. Moreover, we selected videos in which sailing occurred under normal weather conditions. We manually tagged 27 maneuvers to assess our automatic method. The average sensitivity (rate of true positives over total positives) was 72.72%, and three maneuvers were not detected.

On the other hand, by not removing the moments when the assumptions did not hold, the number of false positives increased considerably (from 4 to 22). These false positives can be manually discarded via the visual interface.

The adapted edge-focusing method predicted the exact crossing frame for 20 out of 27 maneuvers. The median offset was 26 frames, with an average of 45 frames. In practice, considering a 30 Hz video, the crossing moment would be off by 1 s, which is negligible in our application. Although more tests need to be conducted using larger amounts of data, this evaluation indicates the potential practical utility of our method.

### Visual analysis framework evaluation

We conducted a user study involving seven coaches to evaluate the visual analysis framework developed for this study. The purpose of the study was to determine whether this tool is considered useful for coaching. The questionnaire consisted of the questions listed in Table 2.

**Table 2:** Questions on the user study

Question	Answer type
Q1: What aspects of this framework, if any, would be useful for coaching?	Open
Q2: What features are you missing in this framework?	Open
Q3: What features are not useful?	Open
Q4: How likely is it that you would use this in coaching?	Likert scale
Q5: How useful, in your opinion, is the timeline with marked intervals/maneuvers in the framework?	1–10 scale

In response to Q1 in Table 2, three coaches pointed out that all aspects of the framework are potentially useful. Others noted that the annotation and marking features were particularly interesting. In response to Q2, two coaches noted that they would like an easy way to share and store the clips. Furthermore, being able to draw annotations on videos and to label the clips were also considered as desirable future features. One coach specifically asked for a zoom feature because “*for the relatively fast sailing boats, it is difficult to stay close during training*.” Although these functionalities were outside the scope of this study, they can be easily added in the future.

The responses to Q3 made us realize that the thumbnails did not give a clear indication of the content. As stated by one coach, “*everything looks the same in the thumbnails and therefore it is not useful*.” Another coach noted that “*you need a way to label/name the thumbnails to be able to distinguish between them*.”

**Fig. 9:**
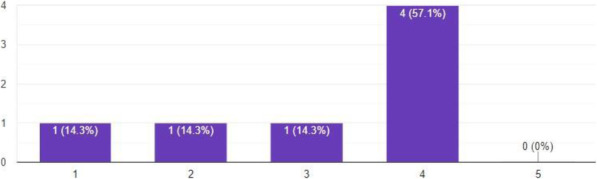
Results of the answers for question Q4: “How likely is it that you would use this in coaching?” Answers were in the form of a 5-point Likert scale

The answers from Q4 lead us to conclude that most coaches would likely use our framework in coaching, as shown in Fig. 9. Coaches who gave low scores chose low scores because of features not covered in this study, such as a zoom feature. On average, 7.43 out of 10 coaches felt that the timeline is a useful feature, based on their responses to Q5.

## Conclusions and future work

We presented a framework to detect maneuvers in a recorded sailing training session and an interface to aid in debriefing. Evaluation of the proposed stabilization method indicates that it increases the visual quality of the video. An immediate future step would be to remove distracting moving edges reported by the coaches participating in our survey.

When our assumptions hold, most maneuvers are automatically detected with high precision of the crossing moment as sailors move from one side of the boat to the other. However, increasing the detection accuracy of the boat and the sailors is still desired. An option is to include sensor data that are sometimes used in training sessions.

The visual interface developed to support debriefing allows coaches to locate interesting moments in the videos, thus avoiding having to search through the entire session manually. The evaluation indicates that our proposed framework provides a promising platform for coaches to analyze training sessions. In the future, we intend to perform further evaluations using more data in the form of training videos.

## Data Availability

Not applicable.

## Abbreviations

ITF: Interframe transformation fidelity
LoG: Laplacian of Gaussian
MSE: Mean-squared error
PPHT: Progressive probabilistic Hough transform
PSNR: Peak signal-to-noise ratio
RIB: Rigid inflatable boat
